# The vernalisation regulator *FLOWERING LOCUS C* is differentially expressed in biennial and annual *Brassica napus*

**DOI:** 10.1038/s41598-019-51212-x

**Published:** 2019-10-17

**Authors:** Sarah V. Schiessl, Daniela Quezada-Martinez, Ellen Tebartz, Rod J. Snowdon, Lunwen Qian

**Affiliations:** 1 Department of Plant Breeding, Justus Liebig University, IFZ Research Centre for Biosystems, Land Use and Nutrition, Heinrich-Buff-Ring 26-32, 35392 Giessen, Germany; 2 Collaborative Innovation Center of Grain and Oil Crops in South China, Hunan Agricultural University, Changsha, 410128 China

**Keywords:** Plant evolution, Plant genetics

## Abstract

Plants in temperate areas evolved vernalisation requirement to avoid pre-winter flowering. In *Brassicaceae*, a period of extended cold reduces the expression of the flowering inhibitor *FLOWERING LOCUS C* (*FLC*) and paves the way for the expression of downstream flowering regulators. As with all polyploid species of the *Brassicaceae*, the model allotetraploid *Brassica napus* (rapeseed, canola) is highly duplicated and carries 9 annotated copies of *Bna.FLC*. To investigate whether these multiple homeologs and paralogs have retained their original function in vernalisation or undergone subfunctionalisation, we compared the expression patterns of all 9 copies between vernalisation-dependent (biennial, winter type) and vernalisation-independent (annual, spring type) accessions, using RT-qPCR with copy-specific primers and RNAseq data from a diversity set. Our results show that only 3 copies – *Bna.FLC.A03b*, *Bna.FLC.A10* and to some extent *Bna.FLC.C02* – are differentially expressed between the two growth types, showing that expression of the other 6 copies does not correlate with growth type. One of those 6 copies, *Bna.FLC.C03b*, was not expressed at all, indicating a pseudogene, while three further copies, *Bna.FLC.C03a* and *Bna.FLC.C09a*b, did not respond to cold treatment. Sequence variation at the *COOLAIR* binding site of *Bna.FLC.A10* was found to explain most of the variation in gene expression. However, we also found that *Bna.FLC.A10* expression is not fully predictive of growth type.

## Introduction

Synchronization of reproduction with favorable environmental conditions is crucial for a species’ survival. For plants, timing of reproduction is mainly governed by flowering time regulation. In temperate climates, the two most important cues to induce flowering are a period of extended cold (the vernalisation pathway) and day length (the photoperiod pathway)^1^. In contrast to biennial or winter forms, annual or spring forms are lacking a functional vernalisation pathway and therefore flower without a period of extended cold.

The vernalisation pathway was found to be a complex regulatory system including transcriptional and epigenetic regulation mechanisms^2^. In *Arabidopsis thaliana*, the model *Brassicaceae*, a single copy of the transcriptional repressor *FLOWERING LOCUS C* (*FLC*) inhibits expression of the central flowering regulator *FLOWERING LOCUS T* (*FT*)^3^. In this state, *FT* repression via *FLC* overrides activating effects from the photoperiod pathway and other activating input signals^4^. FLC also represses the expression of further important flowering genes by binding to the promoter, like *FLOWERING LOCUS D* (*FD*)*, SUPPRESSOR OF OVEREXPRESSION OF CONSTANS 1* (*SOC1*), and *TEMPRANILLO 1* (*TEM1*)^5,6^. While the regulation of many *FLC* targets depends on complex formation with SHORT VEGETATIVE PHASE (SVP), *FT* and *SOC1* expression can be independently regulated by either FLC or SVP^7^. The activating function of FT depends on dimer formation with FD^8^: *FLC* itself is regulated via the vernalisation pathway^2^ and via the autonomous pathway^9^. There is also cross-talk with the photoperiod pathway via the protein SENSITIVITY TO RED LIGHT REDUCED 1 (SRR1)^10^. *FLC* expression is regulated via chromatin conformation, which itself is governed via histone modifications^11^. Genetic and epigenetic regulation processes induced by extended cold stabilize a chromatin state which does not allow transcription of *FLC* mRNA^12^. This inactive state is mitotically, but not meiotically stable; therefore, the next generation starts in a non-vernalized state^3,13^. In the non-vernalized state, *FLC* expression is promoted by the FRI-C, a large protein complex built up by the scaffold protein FRIGIDA (FRI) in interaction with SUPPRESSOR OF FRIGIDA 1 (SUF4)^14^. FRI-C acts to recruit general transcription factors and specific chromatin modification factors to the *FLC* chromatin^14^. Cold induces the transcription of a noncoding antisense transcript from *FLC* called *COOLAIR*, which starts to decrease *FLC* mRNA levels^12,15^. Independently from that, but later, alternative splicing produces the noncoding sense RNA *COLDAIR* from the first intron of *FLC* itself^12,15^. *COLDAIR* recruits Polycomb Group proteins (PcG) proteins forming the PRC2-like complex to the *FLC* chromatin^15^, where they remove activating marks and add repressive marks to the histones in the *FLC* chromatin in interaction with the protein VERNALIZATION INSENSITIVE 3 (VIN3)^16^. This is further supported by the action of the Paf1 complex^17^. Those repressive marks are recognized and bound by the PRC2 complex containing the proteins VERNALISATION 2 (VRN2) and LIKE HETEROCHROMATIN 1 (LHP1) (also known as TFL2), keeping *FLC* repression stable until the next generation^18–20^.

In *A. thaliana*, spring and winter forms have been shown to carry different allelic variants of *FLC, FRI* and *VIN3*^14,21–23^. As the main vernalisation components were found to be conserved in the *Brassicaceae*^24–31^, their paralogs are also candidates for the difference between spring and winter forms in *Brassica* crops. Variation in paralogs of *FLC* was found to be associated to a change in flowering time for *B. rapa*^32–35^, *B. oleracea*^25,36–38^ and *B. napus*^26,28,39–41^. However, these crops have a paleopolyploid origin and were shown to carry several paralogs of *FLC*. The reference genome of *B. napus* carries nine annotated copies of *Bna.FLC*^42^. Early transformation studies revealed that at least five of them are able to delay flowering in *A. thaliana*, but to a varying extent^28^. The strongest effect was observed in plants transformed with *BnFLC1* (*Bna.FLC.A10*)^28^. Later it was discovered that a transposon insertion in the upstream region of *Bna.FLC.A10* is strongly associated to the vernalisation-dependent phenotype^39^. Depending on the genetic background investigated, different copies of *Bna.FLC* were repeatedly found within the confidence intervals of quantitative trait loci (QTL) for flowering time in *B. napus*^43–48^. Previously we found that a *R10P* mutation in the *Bna.FLC.A10* (BnaA10g22080D) copy is a strong candidate for the winter-spring split in a diverse set of *B. napus* accessions^41^. More recently, it was found that the strongest selection signal separating spring and winter type populations was also found at *Bna.FLC.A10*, correlating with differential gene expression^49^. Although different data sets indicate that *Bna.FLC.A10* might be the most decisive copy for vernalisation requirement^28,39,41^, the roles of the other copies remain unclear, mostly because specific primers for some copies could not be developed^29,46^. For example, the most extensive expression study on *Bna.FLC* copies to date was not able to resolve four of the nine copies specifically, and the study was only conducted in a winter and a semi-winter type, but not in a spring type^29^. In the present study, we conducted Reverse Transcription Quantitative Real-Time PCR (RT-qPCR) to measure the specific individual expression levels of all *Bna.FLC* copies. Comparing two winter-type and two spring-type *B. napus* accessions, along with a winter-hardy but not strictly vernalisation-dependent winter accession, we measured *Bna.FLC* expression levels with and without vernalisation, and in different tissues prior to vernalisation. Our results indicate that only *Bna.FLC.A03b, Bna.FLC.A10*, and *Bna.FLC.C02* are differentially expressed between winter and spring type accessions, although tissue-specific differences exist. Comparisons with RNAseq data for a diversity set show that this differential expression is a general difference between winter and spring accessions before vernalisation. We then screened a diverse population of 53 winter and 48 spring accessions for *Bna.FLC.A10* and *Bna.FLC.C02* expression with RT-qPCR and found that only *Bna.FLC.A10* was differentially expressed between winter and spring accessions. There were, however, exceptions in both winter and spring accession, and sequence analysis revealed that this was strongly associated to a sequence variant at the *COOLAIR* binding site and a broken reading frame in exon 1. We conclude that this sequence variant contributed to the difference between biennial and annual *B. napus* forms, whereas their remaining homologs appear to have undergone subfunctionalisation and pseudogenisation after polyploidisation.

## Material and Methods

### Sequence analysis

Genomic sequences were extracted from the respective reference genomes for *B. napus* (the *Darmor-bzh* reference genome, version 4.1^42^), *B. rapa* (the ‘Chiifu-401-42’ reference genome, version 1.5^50^) and *B.oleracea* (the ‘TO1000’ reference genome, version 2.1^51^) using the respective annotation. A further copy of *Bna.FLC* was identified in^41^ and also included. Moreover, we identified four gene copies from an unpublished version of *B. nigra* genome which was made available by the courtesy of I. Parkin. The gene IDs for the single copies can be found in Table S1. The sequence from *A. thaliana* was retrieved from The Arabidopsis Information Resource (TAIR), www.arabidopsis.org, for gene model AT5G10140.1.

Sequence alignments were performed using CLUSTAL multiple sequence alignment by MUSCLE (http://www.ebi.ac.uk/Tools/msa/muscle/, version 3.8) with Default parameters. Based on this alignment, we constructed a Maximum likelihood tree and a neighbor joining tree using bootstrap analysis (100 replicates) using MEGA version 10.0.5. Exon-intron structure was determined by aligning the genomic sequences with the respective cDNA sequences from^42^. SNP variation for the five accessions also used in RT-qPCR was taken from^41^.

Promoter region analysis was performed using MEME (http://meme-suite.org/tools/meme) and JASPAR (http://jaspar.genereg.net/) using motifs for *A. thaliana*^52^.

### Plant material, cultivation and sampling

Two *B. napus* winter accessions (Manitoba, Lisabeth) and two spring accessions (Girasol, Korall) were chosen along with a winter-hardy, vernalization-independent winter accession (Mansholt). Mansholt carries duplications for *Bna.FLC.C09a* and *Bna.FLC.C09b*, while the other accessions did not show copy number variations^53^. The plants were sown in 7 × 7 cm pots in 3 biological replicates per treatment and transplanted to 12 × 12 cm pots 4 weeks after sowing. Cultivation was performed in a greenhouse using a 16/8 h day/night rhythm with 20/17 °C. The first sampling was performed seven weeks after sowing when the plants reach BBCH stage 14 at midday (ZT 7) by cutting off the youngest fully developed leaf. Leaves were frozen in liquid nitrogen and stored at −80 °C until RNA extraction. After the first sampling, one set of plants was brought to the cool room for eight weeks using an 8/16 h day/night rhythm at constant 5 °C. Another set of plants remained in the greenhouse as control plants. After eight weeks of vernalisation, both sets were sampled again at BBCH20.

For the analysis of tissue-specific expression, Manitoba and Korall were grown in three replicates in the greenhouse under the same conditions. Ten weeks after sowing when the plants reach BBCH15, we sampled petioles, developed and emerging leaves separately and kept the samples at −80 °C until RNA extraction.

For the analysis of the time series, Manitoba was grown in 27 replicates in the greenhouse under the same conditions. From an age of 3 weeks up to 8 weeks, we took leaf samples from 3 biological replicates every week. The plants were then subjected to a cold treatment as described above, and we sampled again after 4, 6 and 8 weeks of cold treatment.

For the population screening, 3 biological replicates of 101 accessions (see Supplementary Table 2 for a list of accessions) were sown in quickpot plates and grown for 7 weeks until sampling.

### Primer design, cDNA synthesis and quantitative PCR

Total RNA was extracted using the NucleoSpin miRNA kit (Macherey-Nagel) following manufacturer’s instructions, quantified using Qubit RNA Broad Range on a Qubit fluorimeter and stored at −80 °C until use.

cDNA synthesis was performed using the RevertAid cDNA synthesis kit (ThermoFisher) using 1 µg of total RNA and Oligo-DT primers. cDNA was quantified using the Qubit DNA High Sensitivity kit on a Qubit fluorimeter. Quantitative Real-time PCR was performed on a Real-Time PCR System ViiA7 cycler (Applied Biosystems) in 384-well plates. The reaction mix containing specific primers, template cDNA and FastStart Universal SYBR Green Master mix containing Rox (Roche) was pipetted by a robot (Biomek 4000, Beckman Coulter). As endogenous control, we used ubiquitin. The PCR program was as follows: initial denaturation (94 °C for 2 min), amplification and quantification (40 cycles, 95 °C for 20 sec, 60 °C for 30 sec, 72 °C for 30 sec), and final extension (72 °C for 5 min). A final melting curve was recorded between 55 and 95 °C. PCR efficiency was measured using a pool of all samples in a dilution series of 6 points. All samples were measured in 3 technical replicates. The normalized expression level was determined using the ΔCt method^54^. The primer sequences are shown in Supplementary Table 3. For comparisons between winter and spring, a Student’s t-test was performed, for comparisons of more than one factor, we calculated least significant distances in R using the package agricolae.

### RNAseq analysis

For validation of the results from RT-qPCR, we downloaded a data set from NCBI Sequence Read Archive (SRA, SRP069066) published in^55^. These represent RNAseq data from Illumina HiSeq2500 in single-end mode for a publically available *B. napus* diversity set (ASSYST set^56,57^) for 3 weeks old leaf samples without vernalization. Unfortunately, no biological replicates are given, so the data can only be used for comparison or pooled data analysis. We selected (1) the same 5 accessions which were used in our RT-qPCR experiment for comparison (2) 30 randomly selected spring accessions and 30 randomly selected winter accessions for pooled data analysis (see Supplementary Table 4).

Another publically available data set was downloaded to analyze the time course in gene expression for several vernalisation genes before, during and after cold treatment in the spring cultivar Westar from the NCBI SRA (SRP132445). Each sample was a pool of 6–10 individual plants. We only analyzed time points with two replicates, which were 22 days old plants (before vernalisation), 43 and 64 old plants (during vernalization), and 67 and 72 days old plants (after vernalization). For each time point, both leaves and shoot apex were sampled.

Quality control was performed using FastQC, version 0.10.1 (http://www.bioinformatics.babraham.ac.uk/projects/fastqc/). Accordingly, adapter removal and trimming was performed using Trimmomatic version 0.38^58^ by first removing TruSeq adapters followed by head cropping the first 9 bases. Clean reads were mapped onto version 4.1 of the *B. napus* ‘*Darmor-Bzh’* reference genome using Bowtie2^59^, alignment mode “very-sensitive”. Removal of duplicates, sorting and indexing was carried out with *samtools* version 0.1.19^60^. We then calculated transcripts per million (TPM) using *bedtools* software with multiBamCov^61^ for raw coverage calling, followed by normalization in R (version 3.1.2) according to^62^ for the sum of all exons of every gene. From this, we selected a list of important vernalisation gene copies, and calculated |log_2_(mean(TPM(winter))/mean(TPM(spring))|. Besides *FLC*, we included other important vernalisation regulators (*FRI, SUF4, TFL2, VIN3, VRN2, SVP, SRR1*) and genes directly regulated by vernalisation (*SOC1, FD, FT, TEM1*).

## Results

### Phylogenetic analysis

**Figure 1:**
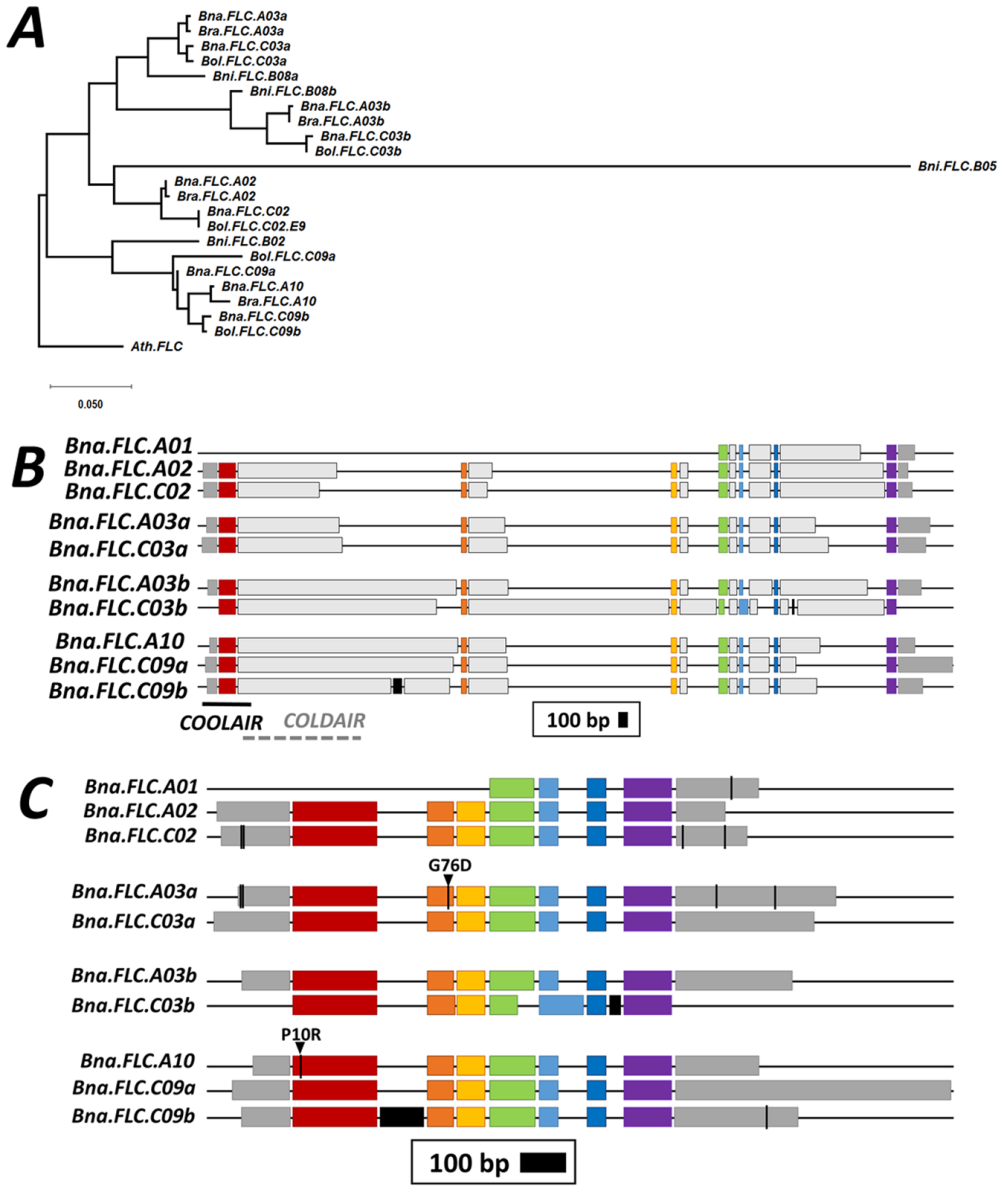
**(A**) Phylogenetic tree constructed with Maximum Likelihood of *Brassica FLC* from three diploid (*B. rapa, B. nigra, B. oleracea)* and one polyploid (*B. napus*) species with *Ath.FLC* as outgroup. cDNA sequences were extracted from the respective reference genomes, the sequence from *A. thaliana* was retrieved from TAIR. Sequence alignment was performed using CLUSTAL multiple sequence alignment by MUSCLE with Default parameters. Based on this alignment, a Maximum Likelihood tree was constructed using MEGA version 10.0.5. (**B)** Full gene structure including UTR (dark grey boxes), introns (light grey boxes) and exons (colored boxes). Black boxes represent extra exons. Box length is proportional to length in bp, the legend shows the length of 100 bp. The solid line in black shows the localization of *COOLAIR*, the dotted line in grey represents the approximate position of *COLDAIR*. **(C)** Exon structure including the position of sequence variants within the 5 accessions used for the initial RT-qPCR. Dark grey boxes represent UTRs, colored boxes represent exons in the same color code than for B, black boxes represent extra exons. Black lines represent a variant, black lines with a black triangle represent a non-synonymous SNP, annotated with the respective amino acid mutation.

The coding sequences of the respective homeologous copies are more related to each other than to their inter-subgenome paralogs (Fig. 1A, Supplementary Fig. 1). This is also evident from the gene structure. Most *Bna.FLC* copies have 7 exons, while the truncated pseudogene on A01 only carries exons 4–7. *Bna.FLC.C03b* and *Bna.FLC.C09b* both carry an additional exon between exons 1/2 and 6/7, respectively (Fig. 1B,C). With the exception of *Bna.FLC.A01*, similar relationships were reported elsewhere^28,29,63^. When comparing all *Bna.FLC* copies with their progenitors from *B. rapa* and *B. oleracea*, there is mostly low divergence between the progenitor and the polyploid (Fig. 1A). The largest distance was found between *Bra.FLC.A02* and *Bna.FLC.A02*, followed by *Bol.FLC.C09b* and *Bna.FLC.C09b*. The only copy without a respective homolog in the progenitors was *Bna.FLC.A01*, suggesting that it has been duplicated after the recent interspecific hybridization leading to *B. napus* (<7500 years ago^42^), most likely from *Bna.FLC.A02* (Supplementary Fig. 2). In contrast, both *Bna.FLC.C09* copies have a closely related homolog in the progenitor species *B. oleracea*, indicating that they have been duplicated earlier. The related *Brassica* species *B. nigra* was found to carry 4 *FLC* copies, however, two of those copies (*Bni.FLC.B02* and *Bni.FLC.B08b*) were truncated in our version of the *B. nigra* reference genome, which possibly is due to incomplete annotation, but might also indicate pseudogenization. The respective copies cluster with the same main four branches than the AC species copies, but more distantly, reflecting the higher divergence of the B genome from the AC genomes. *Bni.FLC.B05* clusters distantly with the A02/C02 clade, *Bni.FLC.B08ab* cluster with the respective A03ab/C03ab clades, and *Bni.FLC.B02* cluster with the A10/C09 clade (Fig. 1A).

**Figure 2:**
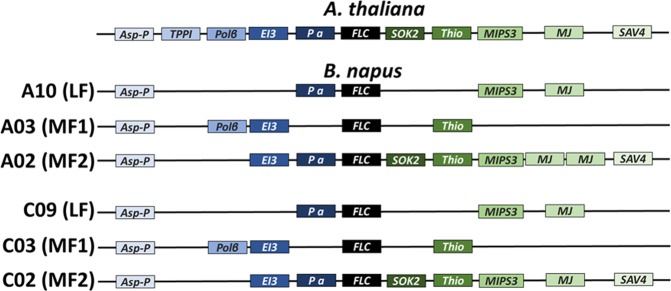
Graphical representation of local synteny for the syntenic *Bna.FLC* copies. The figure shows the gene order in the vicinity of *Ath.FLC, Bna.FLC.A10, Bna.FLC.A03a, Bna.FLC.A02, Bna.FLC.C09a, Bna.FLC.C03a* and *Bna.FLC.C02*, respectively. LF, MF1 and MF2 stand for least fractionated, more fractionated 1 and more fractionated 2, respectively. Synteny was derived from BRAD, gene names were derived from TAIR: Asp-P: aspartyl protease family protein, TPPI: TREHALOSE-6-PHOSPHATE PHOSPHATASE I, Polβ: DNA-directed RNA polymerase subunit beta, EI3: Ethylene insensitive 3 family protein, P a: Pollen Ole e 1 allergen and extensin family protein, SOK2 (no synonym given), Thio: Thioesterase superfamily protein, MIPS3: MYO-INOSITOL-1-PHOSPHATE SYNTHASE 3, MJ: Major facilitator superfamily protein, SAV4: SHADE AVOIDANCE 4.

The *Brassica* A and C (sub)genomes can be subdivided into a least fractionated (LF) and two more fractionated (MF1, MF2) partitions^64^. Using synteny analysis^64^, we found that *Bna.FLC.A10 (BnaA10g22080D)* and *Bna.FLC.C09a (BnaC09g46500D)* are located in LF, *Bna.FLC.A03a (BnaA03g02820D)* and *Bna.FLC.C03a (BnaC03g04170D)* are located in MF1, and *Bna.FLC.A02 (BnaA02g00370D)* and *Bna.FLC.C02 (BnaC02g00490D)* are located in MF2 (Fig. 2). *Bna.FLC.A03b (BnaA03g13630D), Bna.FLC.C03b (BnaC03g16530D)* and *Bna.FLC.C09b (BnaC09g46540D)* are non-syntenic copies, indicating that they arose after the hexaploidization of the common *Brassica* ancestor.

### Analysis of regulatory regions

We compared the sequences of three different regulatory regions using^42^: the promoter regions, intron 1 known to contain the long non-coding RNA *COLDAIR*, and the regions containing the antisense RNA *COOLAIR*.

**Figure 3:**
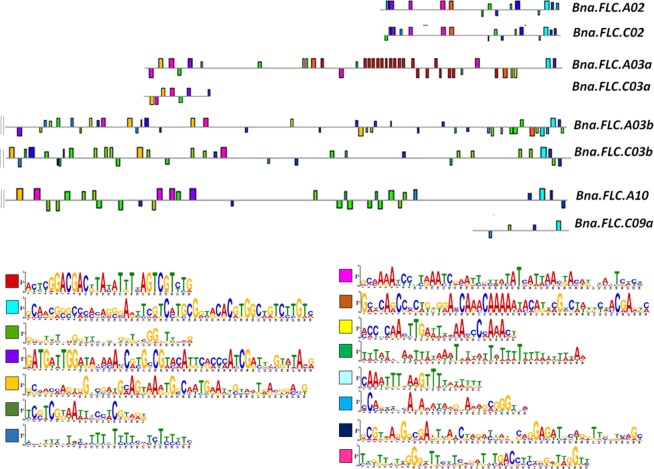
Motif composition of the upstream (promoter) regions of 8 analysed *Bna.FLC* gene copies done by MEME. The promoter region for *Bna.FLC.C09b* was excluded due to missing sequence information. Every motif is represented by a distinct color (see legend).

The promoter regions (the upstream region of the gene extending until the next annotated gene) ranged from 736 bp (*Bna.FLC.C03a*) to 10,928 bp (*Bna.FLC.A03a*). The *Bna.FLC.C09b* promoter region was excluded from the analysis due to a significant amount of missing sequence data. Only a 26 bp motif (consensus **C**M**TGCG**RYR**CAC**R**TGGC**WR**TC**Y**T**S**T**M), located shortly before the 5′UTR of the gene, was highly conserved between the promoter regions of all 8 analysed copies, annotated as MA1359.1, the binding site for the transcription factor *BIGPETAL* (BPEp).The motif also overlapped with the *COOLAIR* binding site. Apart from this, motif search using MEME did not reveal a conclusive pattern. While the promoter regions of *Bna.FLC.A02* and *Bna.FLC.C02* were highly conserved both in length and motif content, the promoter regions of *Bna.FLC.A03a* and *Bna.FLC.A10* were remarkably longer than their C subgenome counterparts and motif composition was different. The promoter regions of *Bna.FLC.A03b* and *Bna.FLC.C03b* were comparable in length, but different in motif composition (Fig. 3). The promoter region of *Bna.FLC.A03b* carries a highly repetitive 29 bp motif (consensus **A**Y**TCGGACGAC**K**TATATTT**H**AGTCGT**Y**TG**) which is specific among all copies. No other motif was specific to a single promoter region. We have also retrieved the respective promoter regions from the diploid *Brassica* species *B. rapa, B. nigra* and *B.oleracea* as well as the promoter region of *A. thaliana*. The alignment shows that conservation is generally low among all 5 species, also given the strong variation in length (736 bp in *Bna.FLC.C03a* – 16946 bp in *Bni.FLC.B08b*). However, some few AT rich regions seem to be fairly conserved (Supplementary Fig. 3). When we aligned subgroups of promoter regions to the *A. thaliana* promoter region, we found that different parts of those regions were conserved within those subgroups (Supplementary Fig. 4). While the A02/C02 subgroup is more conserved towards the beginning and the end of the promoter region, the subgroup A03a/C03a is most conserved in the central part, and the subgroups A03b/C03b and A10/C09a towards the end of the region. The promoter regions from *B. nigra* showed the lowest conservation with *A. thaliana*, while the regions from the A10/C09a group showed the highest conservation with *A. thaliana*. Within *Bna.FLC.A10*, a region with a BLAST hit to the MITE transposon in front of Tapidor-*Bna.FLC.A10* reported by^39^ was found to be unique among the promoter regions. When comparing motif composition in the orthologous copies using MEME excluding *B. nigra*, but including *Bna.FLC.C09b*, we found that motif composition is generally conserved between the orthologous promoter regions. Motif composition was fully conserved for the A02, the A03b, C03b and A10 copies, although distances and order were slightly varying. The A03a, C03a, C09a and C09b promoter regions varied slightly in motif composition, for example, the repetitive sequence found in *Bna.FLC.A03a* was not contained in *Bra.FLC.A03a*, indicating a later origin. Moreover, divergence between homeologs was also absent (A03b/C03b) or low (A02/C02, A03a/C03a), but high for A10/C09ab, indicating stronger divergence in regulatory motifs between the A10 and the C09 copies. The 26 bp motif overlapping with *COOLAIR* was also conserved among almost all species and copies, with the exception of the C03b promoter regions and *Bol.FLC.C02*. (Supplementary Fig. 5). The lengths of intron 1 ranged from 912 bp (*Bna.FLC.C02*) to 2,470 bp (*Bna.FLC.A10*) being considerably longer on A10/C09a and A03b/C03b, but intermediate on C09b. On A02/C02 and A03a/C03a, respectively, the first intron was missing large fragments of 1,000–1,500 bp at the 3′ end. When we aligned those to *A. thaliana COLDAIR* (HG975388.1), alignment was generally poor, making a direct comparison difficult.

Finally, we aligned the sequence of the long non-coding antisense transcript *COOLAIR* (GQ352646.1) to *Bna.FLC* and compared the overlapping regions. *COOLAIR* is supposed to cover parts of the 5′upstream region, exon 1, parts of intron 1, parts of intron 6, exon 7 and parts of the 3′downstream region. However, we were only able to match the 5′upstream region, exon 1 and parts of intron 1 to our sequences. Considerable variation was found at the 5′ end (3′ end of the antisense RNA) with *Bna.FLC.C02* showing an insert of 43 bp, while *Bna.FLC.A10* showed more sequence variation at this site. Both copies on C09, in contrast, were approximately 75 bp shorter at this end (Fig. 1B). While RNAseq data (see below) before vernalisation did not show clear evidence for *COOLAIR* expression, there is a pattern of directional reads in the time series of the spring type at *Bna.FLC.A10*, but coverage was too low for quantification.

### *Bna.FLC* expression levels differentiate before vernalization

**Figure 4:**
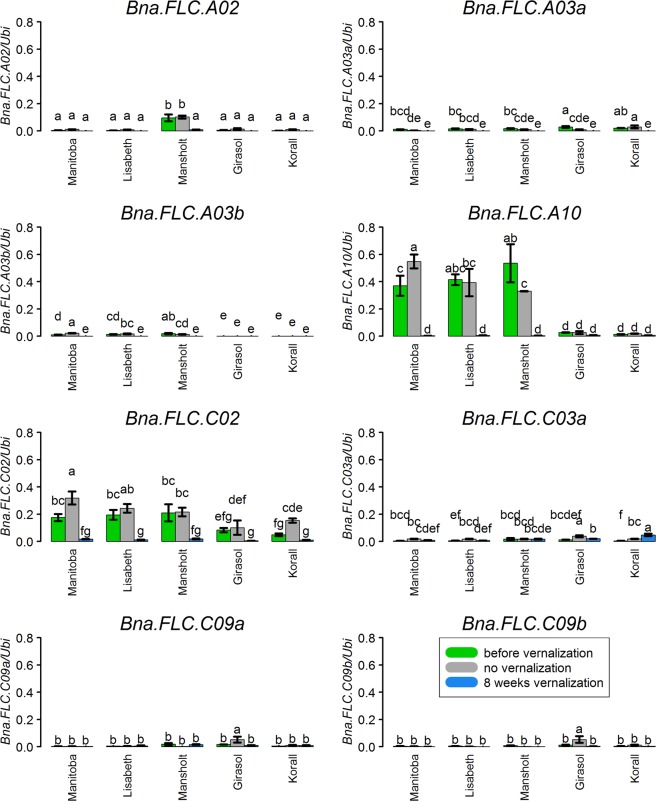
Relative gene expression of all eight expressed *Bna.FLC* copies in leaves before vernalisation 7 weeks after sowing (BBCH 14), and without and with vernalisation 15 weeks after sowing in the accessions Manitoba, Lisabeth (winter-type), Mansholt (winter-hardy, but not vernalisation-dependent), Girasol and Korall (both spring-type). The values were calculated from RT-qPCR using the ΔCt method and represent 3 biological replicates. Whiskers show SEM. “NE” stands for “not expressed”. Small letters represent group by LSD test. The scale is the same for all 3 plots.

We then studied the gene expression patterns of all *Bna.FLC* copies in five different accessions (3 winter and 2 spring cultivars) and found that only eight copies were expressed in leaves seven weeks after sowing (BBCH 14). *Bna.FLC.C03b* was never expressed. The highest expression levels were detected for *Bna.FLC.A10* and *Bna.FLC.C02* in winter accessions. Both copies showed a differential expression pattern between winter and spring accessions, whereby the average ratio of winter/spring expression level was 19.0 for *Bna.FLC.A10* and 2.8 for *Bna.FLC.C02*. The copy *Bna.FLC.A03b* showed no expression in the spring accessions, whereas *Bna.FLC.A02* showed considerably higher expression in Mansholt, a winter type without strict vernalisation requirement (Fig. 4).

We assessed *Bna.FLC* expression in petioles, emerging and developed leaves of 10 week old plants (BBCH 15) before vernalisation in a winter (Manitoba) and a spring accession (Korall) to detect tissue-specific patterns (Supplementary Fig. 6). *Bna.FLC.A03a* and *Bna.FLC.A10* were differentially expressed between winter and spring accessions in all tissues. *Bna.FLC.C02* expression was only significantly different in emerging leaves (p = 0.021) and petioles (p = 0.008), indicating differential expression dynamics in winter and spring material. Additionally, in petioles, *Bna.FLC.A03a* showed a significantly higher expression in Korall (p = 0.037), while *Bna.FLC.C09b* showed a significantly lower expression in Korall (p = 0.019).

For validation, we downloaded available RNAseq data published in^55^. Three week old leaves were sampled before vernalisation for the same accessions we have used here. Only *Bna.FLC.A03b* and *Bna.FLC.A10* were differentially expressed between both growth types. *Bna.FLC.C03b* expression was extremely low. In contrast to RT-qPCR, the expression of *Bna.FLC.A02, Bna.FLC.A03a, Bna.FLC.C02, Bna.FLC.C03a* and *Bna.FLC.C09b* was comparable to *Bna.FLC.A10*, but did not show differential expression between both growth types (Supplementary Fig. 7). Only *Bna.FLC.C09b* had a lower expression level. Those differences may partly be due to the different developmental stage and due to the high sequence similarity of the copies which may confound mapping.

**Figure 5:**
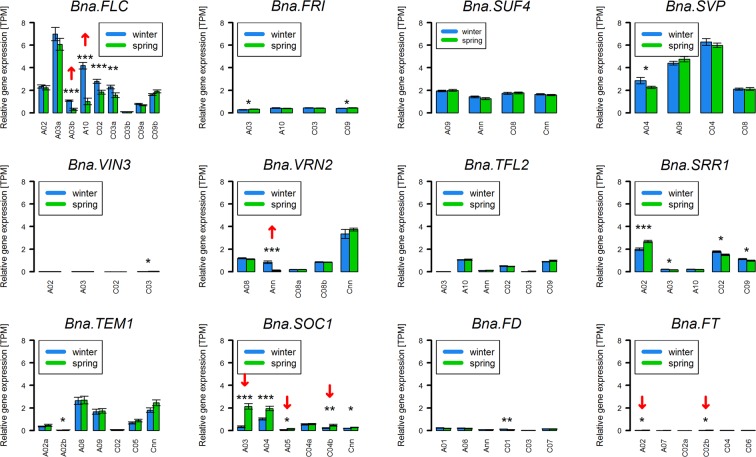
Normalized gene expressed levels (TPM) from RNAseq data for 12 different gene copy groups for genes involved in vernalisation (*FLC, FRI, SUF4, SVP, VIN3, VRN2, TFL2*), affecting *FLC* expression (*SRR1*) or affected by vernalisation (*TEM1, SOC1, FD, FT*) in a diverse set of 30 winter and 30 spring accessions. Whiskers represent standard errors of the mean (SEM). Asterisks show the level of significance based on the Student’s t-test (*p-value < 0.05, **p-value < 0.01, ***p-value < 0.001). Red arrows indicate copies which were strongly (|log_2_(FC)| >1) up or downregulated comparing winter and spring accessions. Arrows pointing upwards indicate augmented expression in winter accessions, arrows pointing downwards indicate lower expression in winter accessions.

We then analyzed a larger data set from the same study containing 30 spring and 30 winter accessions, using all winter accessions as biological replicates for “winter” and respectively so for “spring”. We included other important vernalisation regulators (*FRI, SUF4, TFL2, VIN3, VRN2, SVP, SRR1*) and genes directly regulated by vernalisation (*SOC1, FD, FT, TEM1*). We found 3 gene copies which were strongly (|log_2_(FC)| >1) and significantly (p < 0.05) upregulated in winter accessions. These were *Bna.FLC.A03b, Bna.FLC.A10* and *Bna.VRN2.Ann*. Three copies of the downstream gene *Bna.SOC1* (on A03, A05 and C04) and two copies of the central flowering time gene *Bna.FT* (on A02 and C02) were strongly and significantly downregulated in winter accessions compared to spring accessions. *Bna.VIN3, Bna.FT* and *Bna.FD* were expressed at an extremely low level, as expected (Fig. 5).

### Most *Bna.FLC* copies are downregulated by cold

**Figure 6:**
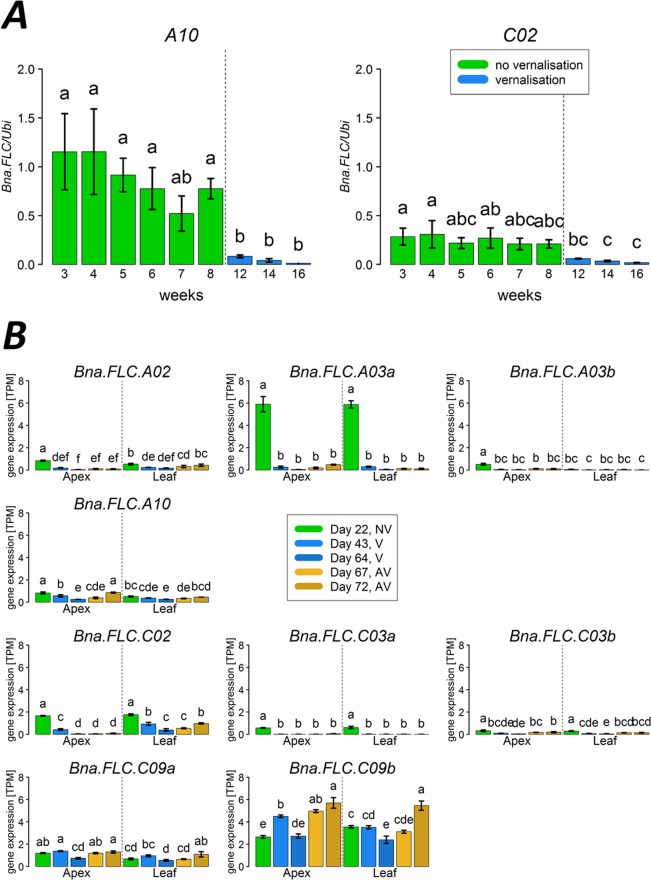
Time series of *Bna.FLC* expression. (**A**) Relative gene expression for *Bna.FLC.A10* and *Bna.FLC.C02* for a time series of 3–16 weeks in the winter cultivar Manitoba. (**B**) Normalized gene expressed levels (TPM) values from RNAseq from a publically available data set for the spring cultivar Westar. Whiskers show SEM. Small letters represent group by LSD test.

Without cold treatment, 15 weeks after sowing (BBCH20), *Bna.FLC* expression patterns did not show a clear pattern. Expression remained mostly constant, although single accessions show slightly decreased expression (*Bna.FLC.A03a, Bna.FLC.A03b, Bna.FLC.A10)* or slightly increased expression *(Bna.FLC.A03b, Bna.FLC.A10, Bna.FLC.C02, Bna.FLC.C03a)*. However, the changes did not affect the differential expression pattern between winter and spring types for *Bna.FLC.A10* (Fig. 4). We also used our primer set to study a time course in the winter type Manitoba for the two copies *Bna.FLC.A10* and *Bna.FLC.C02*. We have sampled independent plants of the same genotype every week from an age of 3 weeks to 8 weeks, when they were subjected to vernalisation. During vernalisation, we sampled at 4, 6 and 8 weeks of vernalisation. The results show clearly that the cold treatment is the only factor influencing gene expression for those copies, while the effect of age can be neglected (Fig. 6A). Longer vernalization seems to further decrease *Bna.FLC* levels, although those effects are only significant for *Bna.FLC.C02*.

After eight weeks of cold treatment (BBCH 20), most *Bna.FLC* copies were downregulated. Exceptions were *Bna.FLC.C03a*, which either did not change or even increased, and both *C09* copies, where downregulation was not significant. Downregulation was generally stronger in winter accessions than in spring accessions, partly due to the higher expression level before vernalisation (Fig. 4).

We also determined *Bna.FLC* and other vernalisation regulators’ expression patterns in a time series in both leaf and apex in a publically available data set from the spring cultivar Westar. For each tissue, we analyzed five time points: 22 days (before vernalization), 43 and 64 days (during vernalization), and 67 and 72 days (after vernalization). The first sampling point was comparable to the sampling point of the other RNAseq data set, and the expression of all copies lay in the same range than the spring accessions from this diversity set. With the exception of *Bna.FLC.C02*, the expression patterns over time were similar in both tissue types. All copies with the exception of *Bna.FLC.C09a* were downregulated during cold. After cold, *Bna.FLC.A10* expression increased again to reach its original state, while both C09 copies even exceeded their original expression (Fig. 6B).

Analyzing the expression patterns of other vernalization regulators in this spring type cultivar, we found that all four copies of *Bna.FRI* got slightly upregulated and all four copies of *Bna.VIN3* got strongly upregulated during cold. *Bna.VIN3* levels returned to their original state after cold, while *Bna.FRI* levels remained almost constant. This indicates that *Bna.VIN3* expression reacts normally in spring types. Interestingly, four out of six *Bna.FT* copies were already fairly well expressed before vernalization, reflecting the spring type behavior, but got completely downregulated during cold treatment and strongly upregulated afterwards. This could possibly be due to a short day length regime in the cold room (Supplementary Figs 8–10).

### Population-wide patterns for *Bna.FLC.A10* and *Bna.FLC.C02*

**Figure 7:**
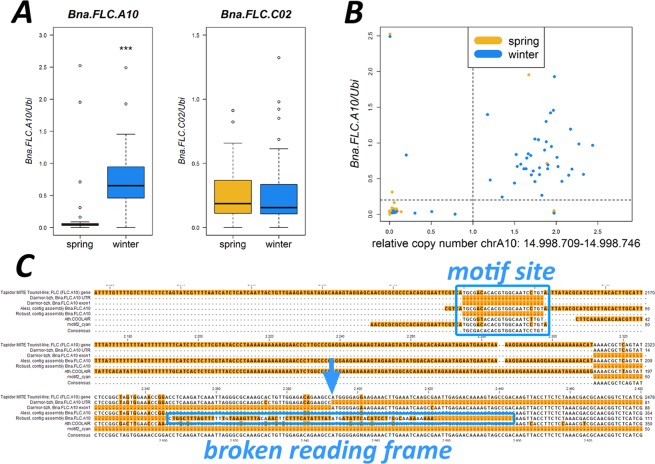
Relationships between the broken reading frame found to be correlating with *Bna.FLC.A10* expression. (**A**) Boxplots for relative gene expression of *Bna.FLC.A10* and *Bna.FLC.C02* in 48 spring and 53 winter accessions in leaves of 7 week old plants. (**B**) Relative copy number (RCN) of the region containing the sequence variant as a proxy for the presence of the sequence variant plotted against *Bna.FLC.A10* relative gene expression. The dotted lines separate “low” and “high” RCN/ gene expression. (**C**) Sequence alignment of the unaffected (accession Alesi) and affected (accession Robust) contig assembly with the Darmor-bzh-5′UTR, the Darmor-bzh exon 1, the Tapidor full length sequence from^39^, the *A. thaliana COOLAIR* sequence and the sequence of the conserved 26 bp motif (indicated by blue frame). The site of the broken reading frame is also indicated. The blue arrow shows the ATG start site.

We then wanted to assess if the two most expressed copies (*Bna.FLC.A10* and *Bna.FLC.C02*) could be associated to growth type on a population-wide scale. For this, we have screened a large set of 101 accessions (53 winter and 48 spring accessions) for gene expression with RT-qPCR. We found that *Bna.FLC.A10* gene expression was highly significantly different between winter and spring accessions (p < 1.8 × 10^−7^), but *Bna.FLC.C02* gene expression was not. For *Bna.FLC.A10*, although the overall pattern was clear, some exceptions were observed in both winter and spring accessions (Fig. 7A). We found four spring accessions with a high *Bna.FLC.A10* expression, while we found ten winter accessions with low *Bna.FLC.A10* expression. Although the population has been selected to reflect both early and late flowering accessions, there is no bias towards *Bna.FLC.A10* expression in early or late flowering accessions. To understand how vernalisation may be upheld or circumvent, we checked the RNAseq diversity set and found four accessions with unusual *FLC* pattern in both the spring and the winter pool. We found that in a winter background, accessions with lower *Bna.FLC.A10* have increased *Bna.SOC1* expression for a copy on A03 (still lower than spring types) and a copy on A05 (higher than spring types) and increased expression of *Bna.FT.A02* (but still considerably lower than spring types). At the same time, they have less *Bna.VIN3.A02*, even lower than spring accessions. In a spring background, we found increased expression of *Bna.TEM1.C02* (higher than winter), but decreased expression of *Bna.FT.A02* (even lower than in winter), *Bna.VIN3.C02* (to 0) and *Bna.TFL2.C03* (approximately winter level).

To understand which causes could be responsible for this pattern, we went back to the resequencing data published in^41^. Here, we realized that a short fragment encompassing the R10P SNP was only covered by non-uniquely mapping reads in certain accessions, while perfectly covered with uniquely mapping reads in others. This points to a previously undiscovered short InDel. InDels of a size between 20–50 bp are hard to detect with short read sequencing, because the respective reads will be too divergent to be mapped. Therefore, we have extracted the reads from all *Bna.FLC* copies and reassembled them for both an affected and a non-affected accession. Aligning the resulting contigs with both the Tapidor and the Darmor-bzh version reveals that the start of the coding sequence is severely distorted in the affected accession, effectively breaking the open reading frame. The start codon is still present, but both the 5′UTR and the first exon are completely divergent from the non-affected sequence. This also affects the presence of the conserved 26 bp motif in front of the 5′UTR, which is missing in the affected accession, and the *COOLAIR* binding site. The first exon misses a fragment of 23 bp, representing a frameshift deletion (Fig. 7C). To assess this pattern for the total population, we took normalized coverage in the respective region (38 bp) as a proxy for this pattern and found that this is almost fully (two exceptions) explaining the observed expression pattern, indicating that either promoter variation or a broken reading frame of *Bna.FLC.A10* contributes to the winter-spring split (Fig. 7B).

## Discussion

### Regulatory diversification among *Bna.FLC* copies predates interspecific hybridization

**Figure 8:**
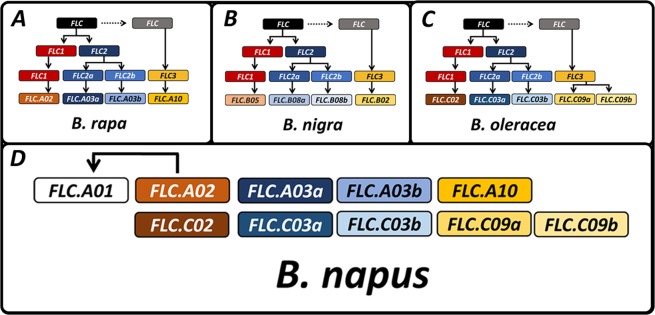
Proposed evolutionary path for *Bna.FLC*. The model was build based on synteny analysis and phylogenetic analysis. Pseudogenes were identified using protein sequence prediction and gene expression analysis. Gene copies are represented as colored rectangles. White color indicates a putative pseudogene. Arrows indicate ancestry. (**A–C**) Proposed evolutionary paths for the diploid *Brassica* species *B. rapa*, *B. nigra* and *B. oleracea*, respectively. (**D**) Copy configuration for the allotetraploid *B. napus*.

*FLC* is a MADS-box transcription factor. The MADS-box genes represent a family of transcription factors regulating different developmental transitions, including flowering and fruit development^65^. Duplication and diversification played an important role in the evolution of the different clades in the MADS-box family^65,66^. One of the most important sources of duplication is polyploidy. Here, we analysed the expression patterns of all *FLC* homologs in *B. napus*, a recent allotetraploid which arose from two mesohexaploid ancestors. Due to the complex genetic history of *B. napus* including polyploidy and gene loss, a copy number between 4 and 5 is expected for single-copy *A. thaliana*^27,67^. *Bna.FLC*, however, was not affected by gene loss, but has rather undergone copy number amplification, as 10 loci could be identified as homologs of *Ath.FLC*. Based on our analysis, we now propose an evolutionary path for *Bna.FLC* (Fig. 8). Therein, we conclude that the duplication of the A03 and C03 copies predates the differentiation of *B. rapa, B.nigra* and *B. oleracea*, while the duplication of the C09 copies occurred after the AC split during *B. oleracea* speciation. *Bna.FLC.C03b* is considered to be a pseudogene, as we and others^29^ never observed expression. The predicted exon structure and protein of *BnaFLC.C03b* differs from the other expressed copies (Fig. 1B,C), and its promoter misses conserved motifs which seem to be important for expression. We hypothesise that *BnaFLC.A01* occurred after the allopolyploidization, probably from the closest homolog *Bna.FLC.A02*, as no close homolog exists in any of the progenitors. The copies on A10 and C09 appear to be most conserved with *A. thaliana*, we therefore propose that the ancestor of this group is less recent and came from the second polyploidization step of the *Brassica* ancestor at the transition from the tetraploid to the hexaploid stage. Indeed, *Bna.FLC.A10* and *Bna.FLC.C09a* are both located within the least fractionated parts of the subgenomes^64^, the youngest part of each subgenome. Given the observation that the copies which were most expressed in each subgenome are not homeologous to each other, subfunctionalisation is likely to have happened during the progenitors’ evolution. This is supported by the high conservation of motif composition in the promoter regions of orthologous copies, indicating that evolution of regulatory elements predates the interspecific hybridization.

### Different cold responsiveness points to subfunctionalisation of different *Bna.FLC* copies

*B. napus* is grown in diverse climates and therefore has developed different growth types. Due to the low genetic diversity within the growth type gene pools^68^, suitable markers for growth type would help to speed up introgression breeding and allow for better and more effective adaptation. We therefore aimed to study the gene expression patterns of the most prominent candidate gene for vernalisation. A functional vernalisation system prevents pre-winter flowering in winter rapeseed, but is generally missing in spring rapeseed. *FLC* copies have been proposed as candidate genes for flowering time in previous QTL studies and GWAS, both in *B. napus* and in the progenitor species *B. oleracea* and *B. rapa*. In the progenitor species, *Bra.FLC.A10* and *Bol.FLC.C02* have been found to underlie QTL for flowering time^25,32,33,36^. In *B. napus*, *Bna.FLC.A10* has been proposed by different authors to be the most important vernalisation gene copy^28,39,41,49^. Others also found associations on C02 and A03 in different material^29,43,46^. Recently, it was shown that promoter variation for *Bna.FLC.A10* was linked to strong selection signatures between winter and spring material^49^, but their data also show high variation in *Bna.FLC.A10* expression especially in winter accessions. *B. napus*, however, has 9 annotated gene copies and therefore underwent gene amplification rather than gene loss. We were therefore interested in the gene expression patterns of all other copies in *B. napus*. Studies on gene expression of other *Bna.FLC* copies have been carried out by other authors. Tadege *et al*.^28^ was the first to qualitatively study *Bna.FLC* expression. They found that *At.FLC* delayed flowering in spring rapeseed, and that some copies of *Bna.FLC* also delayed flowering in *A. thaliana*, with *Bna.FLC.A10* having the strongest effect. They also showed a strong difference between total *Bna.FLC* level between two spring and two winter accessions by Northern blotting, all aligning well with our findings. Moreover, they found *Bna.FLC.A10* being more expressed in stems than in leaves, a finding we could not confirm. Hou *et al*.^39^ studied a large BC_5_F_2_ population derived from a cross between Tapidor (a winter type) and Ningyou7 (a semi-winter type). They could show that *Bna.FLC.A10* downregulation was much quicker in semi-winter than in winter type, but original *Bna.FLC.A10* expression was high in both accessions, indicating that the mechanism separating semi-winter and winter might be different from the mechanism separating winter and spring, as we have found the *Bna.FLC:A10* expression is constantly low in spring types even before vernalization (Figs 4, 6). The authors found a 621 bp MITE insertion upstream of *Bna.FLC.A10* being associated to this lower responsiveness. They did not find allelic variance between the winter and the semi-winter type and concluded that the difference between winter and semi-winter was entirely due to the expression difference. Raman 2016 studied *Bna.FLC* expression unspecifically for the pairs *FLC1* (*Bna.FLC.A10/C09ab*), *FLC2* (*Bna.FLC.A02/C02*) and *FLC3* (*Bna.FLC.A03a/C03a*) in Australian and Japanese material^46^ and found that *FLC2* expression was correlating to vernalization response, while *FLC1* and *FLC3* did not. However, we could not find a study showing expression patterns for all *Bna.FLC* copies in both winter and spring accessions to allow conclusions on possible subfunctionalisation. Therefore, we designed specific primers for all *Bna.FLC* copies and use them to test gene expression both in winter and spring accessions. We found strongly varying expression patterns in respect to expression level, response to cold and specificity to growth type, indicating a high degree of subfunctionalisation. The differential expression of *Bna.FLC.A10* between winter and spring as found by others^49^ has been confirmed. At the same time, we also found *Bna.FLC.A03b* differentially expressed between winter and spring material. *Bna.FLC.C02*, however, was found to be differentially expressed in emerging leaves, but not in developed leaves, and population screening showed either a small (RNAseq data) or no difference (RT-qPCR). Moreover, the copy was shown to be deleted both in winter and spring accessions^53^, which in most cases also correlated with the expression pattern (data not shown), but had obviously no effect on life cycle characteristics. Both copies on C09 and partly *Bna.FLC.C03a* were found not to respond to cold, and *Bna.FLC.C03b* seems to be pseudogene as reported by others^29^. *Bna.FLC.A02, Bna.FLC.A03a* and *partly Bna.FLC.C03a* are expressed and react to cold, but do not show differences in expression between winter and spring. This patterns indicates that *Bna.FLC.A10* is indeed the major source of variation in vernalisation requirement, while *Bna.FLC.A03b* and *Bna.FLC.C02* might still be supportive.

### Accessions with low *Bna.FLC.A10* expression have a broken reading frame

When screening a population of 101 accessions for *Bna.FLC.A10*, we could show that winter accessions express *Bna.FLC.A10* significantly stronger than spring accessions. However, our data along with others^39,49^ show that there are winter accessions with a very low *Bna.FLC.A10* level, which still need vernalisation to flower, and spring accessions with high *Bna.FLC.A10* levels which can flower without. Reassessment of deep-sequencing data on this copy allowed us to detect a sequence variant at the junction of the 5′UTR and exon 1 which was almost fully explanatory for *Bna.FLC.A10* level. The variant overlapped with the binding site of the regulatory antisense RNA and also with a highly conserved 26 bp promoter motif shortly before the 5′UTR. We are therefore now able to describe the nature of the promoter variation which has been proposed by others before^39,49^. This also aligns with other findings, for example, the shorter 3′ end of *COOLAIR* in both C09 copies which do not react to cold. A similar mechanism has been found in *A. thaliana*, where variation in intron 1 caused a different splicing behavior of *COOLAIR*, increasing *FLC* expression^69^. While we now are able to explain the expression patterns in *Bna.FLC.A10*, accessions with unusual expression patterns indicate that *Bna.FLC.A10* might be important, but not necessary to define the life cycle characteristics of winter and spring rapeseed. In winter accessions with low *Bna.FLC.A10*, we see upregulation of some downstream targets like *Bna.SOC1* and *Bna.FT*. In spring accessions with high *Bna.FLC.A10*, we also see a decrease in downstream targets. This could indicate that those exceptions might also have a respective phenotype, but this is not the case, as both early and late flowering accessions are among those exceptional accessions. Therefore, some other genes seem to compensate the effect, but obviously not a different *Bna.FLC* copy. In spring accessions, *Bna.TEM1.C02* is possibly serving this task, as it is known to be an early suppressor of *FT* in *A. thaliana*^70^. Apart from those exceptions, the analysis of the RNAseq data has shown that *Bna.VRN2.Ann* is also downregulated in spring accessions. Other vernalisation genes are not or not strongly differentially expressed at this time point. At the same time, copies of important downstream targets like *Bna.FT* (A02, C02) and *Bna.SOC1* (A03, A05, C04) are significantly downregulated in winter accessions, indicating that the vernalisation system indeed is active, although *Bna.FT* expression levels remain low in spring accessions, probably due to non-inductive photoperiod.

## Conclusions

Our data support earlier findings showing that *Bna.FLC.A10* is the most important copy of *FLC* regulating vernalisation in winter-type *B. napus*. Moreover, we were able to show that this expression pattern is linked to a sequence variant at the *COOLAIR* binding site. However, *Bna.FLC.A10* expression level is not fully predictive of growth type, and more research needs to be done on other factors involved in vernalisation. Accessions showing exceptional patterns of *Bna.FLC.A10* might be useful for such an approach, as they represent functional mutants in the respective background. Here, those mutants have shown that other *Bna.FLC* copies do not compensate lack or overexpression of *Bna.FLC.A10*. The question remains why *Bna.FLC* copy number increased so strongly if additional homologs are not needed for vernalisation. The answer may lie in the assumption of different regulatory roles besides vernalisation. Our finding that some copies are obviously no longer cold-responsive suggests this to be the case, and is reflected by the large variation in putative regulatory regions between the copies. Together these findings indicate beginning gene copy subfunctionalisation, implying ongoing functional diversification in this recent allopolyploid.

## Supplementary information

Supplementary information

## Data Availability

All primer sequences are available from Table S3.
