# Supplementary material for: The vernalisation regulator FLOWERING LOCUS C is differentially expressed in biennial and annual Brassica napus
Source: Sci Rep. 2019 Oct 17;9:14911. doi: 10.1038/s41598-019-51212-x (PMC6797750; doi:10.1038/s41598-019-51212-x)
Supplement: Supplementary file 1 — Supplementary information [file 41598_2019_51212_MOESM1_ESM.docx]

**SUPPLEMENTARY INFORMATION**

for

The vernalisation regulator *FLOWERING LOCUS C* is differentially expressed in biennial and annual *Brassica napus*

Authors: Sarah V. Schiessl^1*^, Daniela Quezada-Martinez^1*^, Ellen Tebartz^1^, Rod J. Snowdon^1^, Lunwen Qian^2^

Affiliation: ^1^ Department of Plant Breeding, Justus Liebig University, IFZ Research Centre for Biosystems, Land Use and Nutrition, Heinrich-Buff-Ring 26-32, 35392 Giessen, Germany

^2^ Collaborative Innovation Center of Grain and Oil Crops in South China, Hunan Agricultural University, Changsha 410128, China

Contact e-mail addresses:

DQM: [Daniela.quezada@agrar.uni-giessen.de](mailto:Daniela.quezada@agrar.uni-giessen.de)

ET: ellen.tebartz@agrar.uni-giessen.de

RJS: [Rod.snowdon@agrar.uni-giessen.de](mailto:Rod.snowdon@agrar.uni-giessen.de)

LQ: qianlunwen@163.com

Corresponding authors: Sarah Schiessl, E-mail: [sarah-veronica.schiessl@agrar.uni-giessen.de](mailto:sarah-veronica.schiessl@agrar.uni-giessen.de), Phone: 0049-641-9937432, ORCID: <https://orcid.org/0000-0003-4372-858X>

Lunwen Qian, E-mail: [qianlunwen@163.com](mailto:qianlunwen@163.com), Phone: 0086-731-84618076, ORCID: https://orcid.org/0000-0002-5426-1340

* These authors contributed equally to the work.

**Table S1**: Gene IDs for all *B.napus* FLC homologs in this study along with their respective orthologs in *B. rapa* and *B.oleracea and with recently used geneIDs from other studies*. As different databases use two different gene IDs, we provide both.

**Table S2:** List of selected accessions for the RT-qPCR screening for Bna.FLC.A10 and Bna.FLC.C02 gene expression for 53 winter and 48 spring accessions. FT stands for flowering time.

| **ID** | **Accession Name** | **type** | **FT** |
| --- | --- | --- | --- |
| ASSYST003 | Robust | winter | late |
| ASSYST005 | Pirola | winter | early |
| ASSYST011 | Picasso | winter | late |
| ASSYST012 | Lord | winter | early |
| ASSYST018 | Montego | winter | early |
| ASSYST022 | Missouri | winter | late |
| ASSYST027 | Licapo | winter | late |
| ASSYST029 | Idol | winter | early |
| ASSYST031 | BRISTOL | winter | early |
| ASSYST032 | Lirajet | winter | late |
| ASSYST034 | Lipid | winter | early |
| ASSYST044 | Laser | winter | late |
| ASSYST046 | Smart | winter | early |
| ASSYST047 | Roxet | winter | late |
| ASSYST052 | SWGospel | winter | early |
| ASSYST053 | Verona | winter | late |
| ASSYST054 | Tenor | winter | early |
| ASSYST056 | Musette | winter | early |
| ASSYST057 | Kvintett | winter | late |
| ASSYST060 | Viking | winter | early |
| ASSYST064 | Baros | winter | early |
| ASSYST068 | Zephir | winter | late |
| ASSYST069 | SLM 0413 | winter | early |
| ASSYST071 | LSF 0519 | winter | late |
| ASSYST072 | Beluga | winter | early |
| ASSYST077 | Duell | winter | early |
| ASSYST079 | Jessica | winter | late |
| ASSYST082 | Prince | winter | early |
| ASSYST088 | KW1519 | winter | late |
| ASSYST113 | Samourai | winter | late |
| ASSYST120 | Darmor | winter | early |
| ASSYST123 | Falcon | winter | late |
| ASSYST132 | Anja | winter | late |
| ASSYST134 | Bolko | winter | late |
| ASSYST135 | Brink | winter | early |
| ASSYST142 | Erra | winter | late |
| ASSYST143 | Gross-Luesewitzer | winter | late |
| ASSYST145 | Hokkai 3-Go | winter | early |
| ASSYST147 | Jantar | winter | late |
| ASSYST149 | Krapphauser | winter | late |
| ASSYST150 | Kromerska | winter | early |
| ASSYST151 | Librador | winter | late |
| ASSYST154 | Liporta | winter | early |
| ASSYST155 | Lirafit | winter | late |
| ASSYST157 | Madora | winter | late |
| ASSYST162 | Moldavia | winter | early |
| ASSYST168 | Ramses | winter | early |
| ASSYST171 | Skziverskij | winter | early |
| ASSYST172 | Slovenska Krajova | winter | early |
| ASSYST175 | Start | winter | late |
| ASSYST180 | V8 | winter | late |
| ASSYST182 | 25629-3 | winter | late |
| ASSYST203 | Taisetsu | winter | late |
| ASSYST244 | COMET | spring | early |
| ASSYST245 | CRESOR | spring | late |
| ASSYST253 | NIKLAS | spring | early |
| ASSYST258 | N01D-1330 | spring | early |
| ASSYST265 | KROKO | spring | late |
| ASSYST267 | LINETTA | spring | late |
| ASSYST272 | Alku | spring | early |
| ASSYST274 | Ceska Krajova | spring | late |
| ASSYST282 | Svalöf’s Gulle | spring | early |
| ASSYST284 | Tribute | spring | early |
| ASSYST285 | Wesway | spring | early |
| ASSYST290 | Ability | spring | early |
| ASSYST291 | Campino | spring | early |
| ASSYST292 | Clipper | spring | early |
| ASSYST299 | Larissa | spring | early |
| ASSYST300 | Magma | spring | early |
| ASSYST307 | Tribune | spring | early |
| ASSYST311 | Q2 | spring | early |
| ASSYST312 | Adamo | spring | late |
| ASSYST313 | Altex | spring | early |
| ASSYST314 | Andor | spring | early |
| ASSYST319 | Concord | spring | late |
| ASSYST321 | Czyzowska | spring | early |
| ASSYST322 | Daichousen (fuku) | spring | late |
| ASSYST327 | Futura | spring | late |
| ASSYST329 | Giant Xr707 | spring | late |
| ASSYST330 | Gisora | spring | late |
| ASSYST346 | Liraspa | spring | late |
| ASSYST349 | Loras | spring | early |
| ASSYST352 | Masora | spring | late |
| ASSYST353 | Miyauchi Na | spring | late |
| ASSYST354 | Nakate Chousen | spring | late |
| ASSYST357 | Olivia | spring | late |
| ASSYST361 | Omega | spring | late |
| ASSYST363 | Orpal | spring | late |
| ASSYST365 | Pera | spring | late |
| ASSYST367 | Pobeda | spring | early |
| ASSYST369 | Pura | spring | early |
| ASSYST373 | Rucabo | spring | late |
| ASSYST374 | Sabine | spring | late |
| ASSYST380 | Sv 75716 | spring | early |
| ASSYST383 | Triton | spring | early |
| ASSYST384 | Uranus | spring | late |
| ASSYST385 | Valecovska | spring | early |
| ASSYST389 | Wase Chousen | spring | late |
| ASSYST393 | Wesroona | spring | early |
| ASSYST397 | Ww1289 | spring | late |
| ASSYST399 | Zairai Chousenshu | spring | late |

Table S3: List of primers used for qPCR. The primers are shown in 5’-3’ direction. The first primer sequence is always forward, the second primer is reverse. The annealing temperature was 60 °C.

| **Target** | **Sequence 5’-3’** | **Fragment size (bp)** |
| --- | --- | --- |
| *Bna.FLC.A02* | CGGCGAGAGTTGAAACCGAAT | 74 |
|  | CTTCCCATGGCTTCTGTCTCC |  |
| *Bna.FLC.A03a* | TCATGGCGAAGAAGCCTACC | 105 |
|  | ACAACCTCTATGCGTTGTGGA |  |
| *Bna.FLC.A03b* | CCTGTAACTCTCCCGCTGCTT | 124 |
|  | AGTAAAGGTGGTTAATTAAGCTGCGA |  |
| *Bna.FLC.A10* | GCCGAAGCTGATAATATGGATGTC | 70 |
|  | GTGGGAGCGTTACCGGAAGA |  |
| *Bna.FLC.C02* | GAGAGCTGAACCGAACCGAA | 143 |
|  | TTGCGTCGTTTGGAGAAGGT |  |
| *Bna.FLC.C03a* | TGAGTTTGCTTGTGTGTTCTTCC | 70 |
|  | GTTGCAAATTAGACGGCTGGC |  |
| *Bna.FLC.C03b* | ATCTGTCGGGCTTCTCGTTG | 96 |
|  | ACCATGTCGGGTGTTCCATC |  |
| *Bna.FLC.C09a* | GATTTATGCAGCGTGGGACAGT | 98 |
|  | GAGGCTCGCTCAGGTTTGGTA |  |
| *Bna.FLC.C09b* | TCGAGAGGCTTCGGGTGTAA | 123 |
|  | GATACACAAGCAAGCTCGAAGTGA |  |
| *Ubiquitin* | CTTCTTCGGCCTCAACTGGTT | 101 |
|  | GAAGATGATCTGCCGCAAGTGT |  |

**Table S4:** List of selected accessions for the RNAseq analysis of 60 diverse accessions.

**
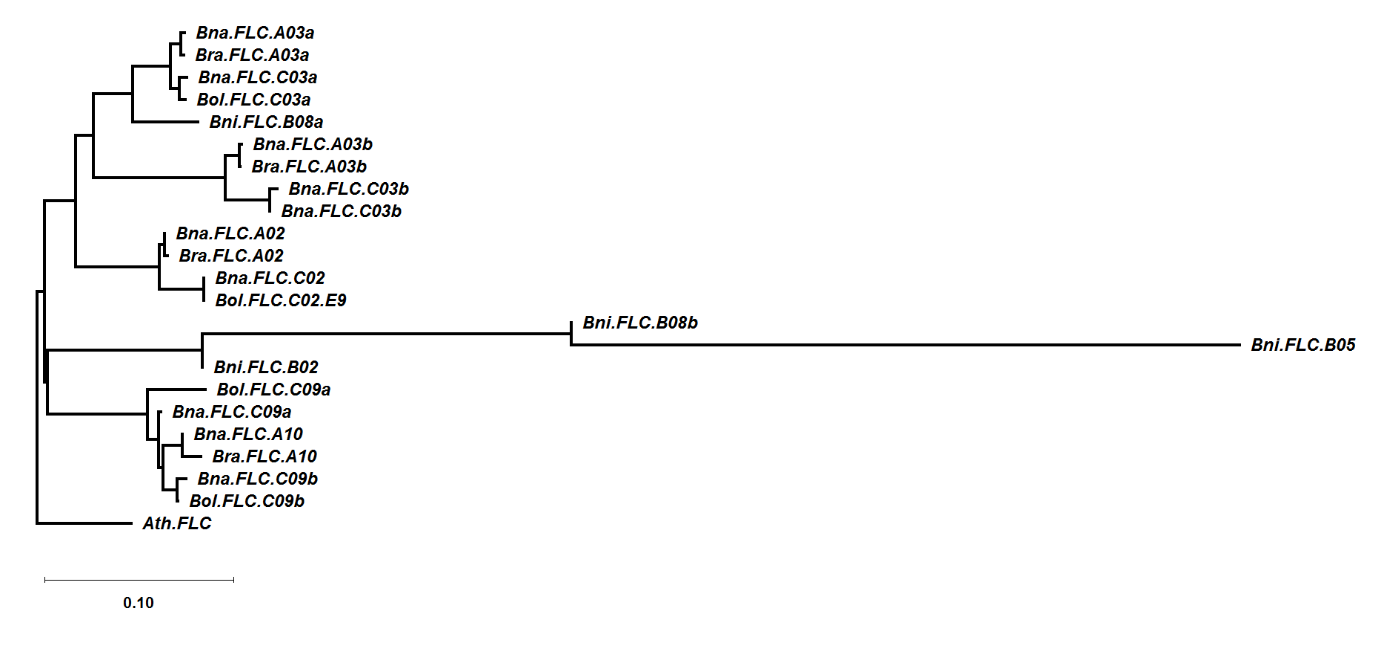
**

**Figure S1:Neighbor-joining tree for all 23 genomic sequences for *FLC* homologs from *Brassica rapa (Bra), Brassica nigra (Bni); Brassica oleracea (Bol)* and *Brassica napus (Bna)* with *Ath.FLC* as outgroup.**

**
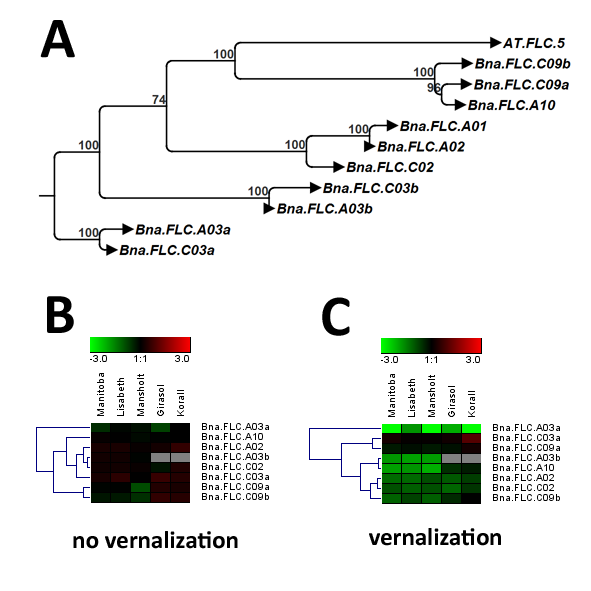
**

**Figure S2: (A) Neighbor-joining tree for all 19 genomic sequences for *FLC* homologs from *Brassica rapa (Bra), Brassica oleracea (Bol)* and *Brassica napus (Bna)* with *AT.FLC* as outgroup.**

**
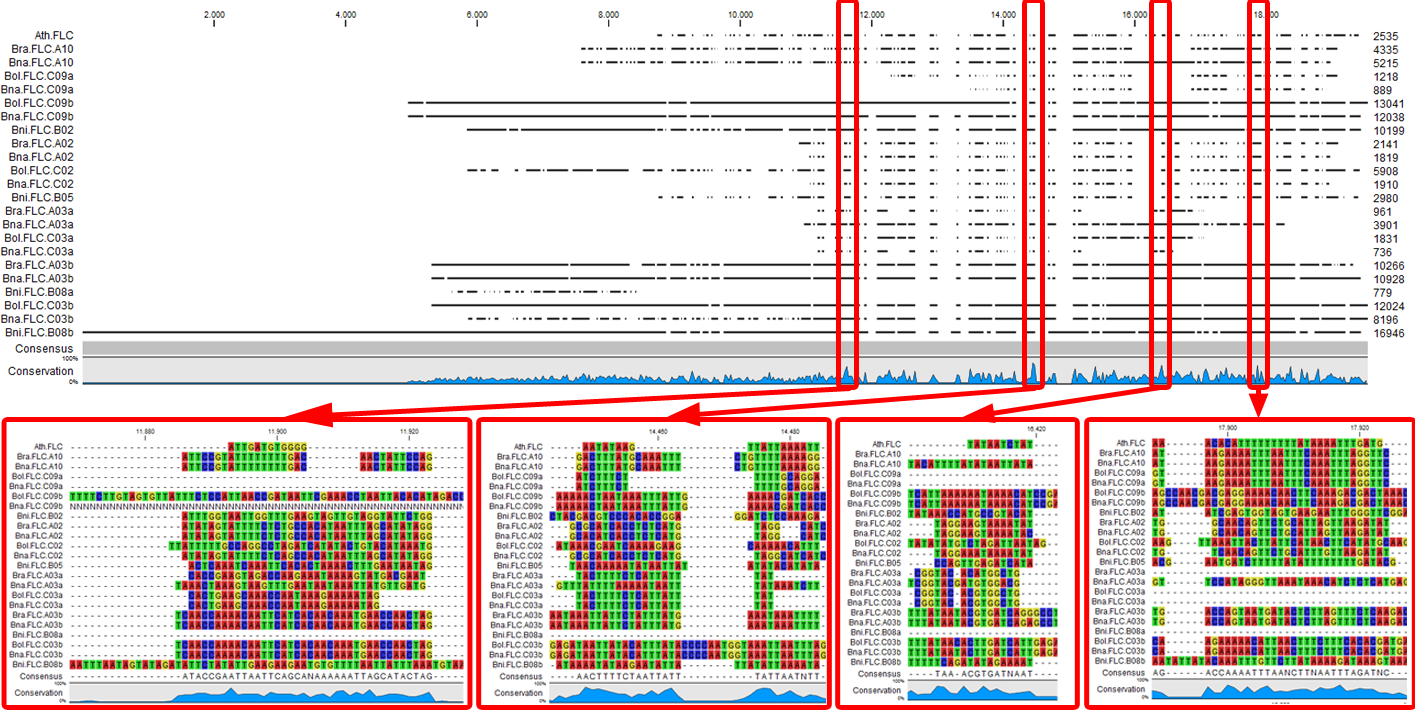
**

**Figure S3:Sequence alignment of all 23 *FLC* promotor regions from 5 species (*B. rapa, B. nigra, B. oleracea, B. napus, A. thaliana*). Highly conserved regions are shown in zoom-in boxes.**

**
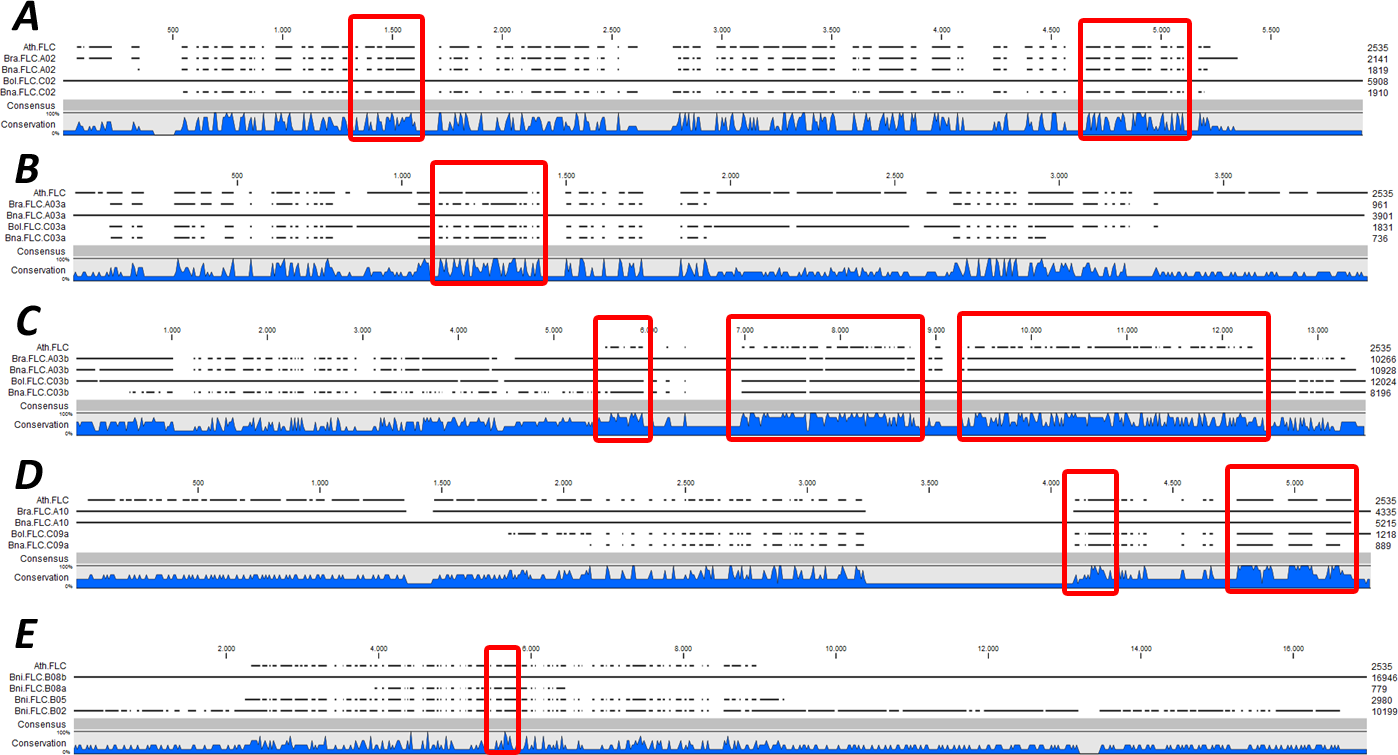
**

**Figure S4: Sequence alignments performed with ClustalW for different groups of promotor regions, each aligned to the *A. thaliana* promotor region. Conserved regions are highlighted in red. (A) A02/C02 (B) A03a/C03a (C) A03b/C03b (D) A10/C09a (E) *B. nigra* promotor regions.**


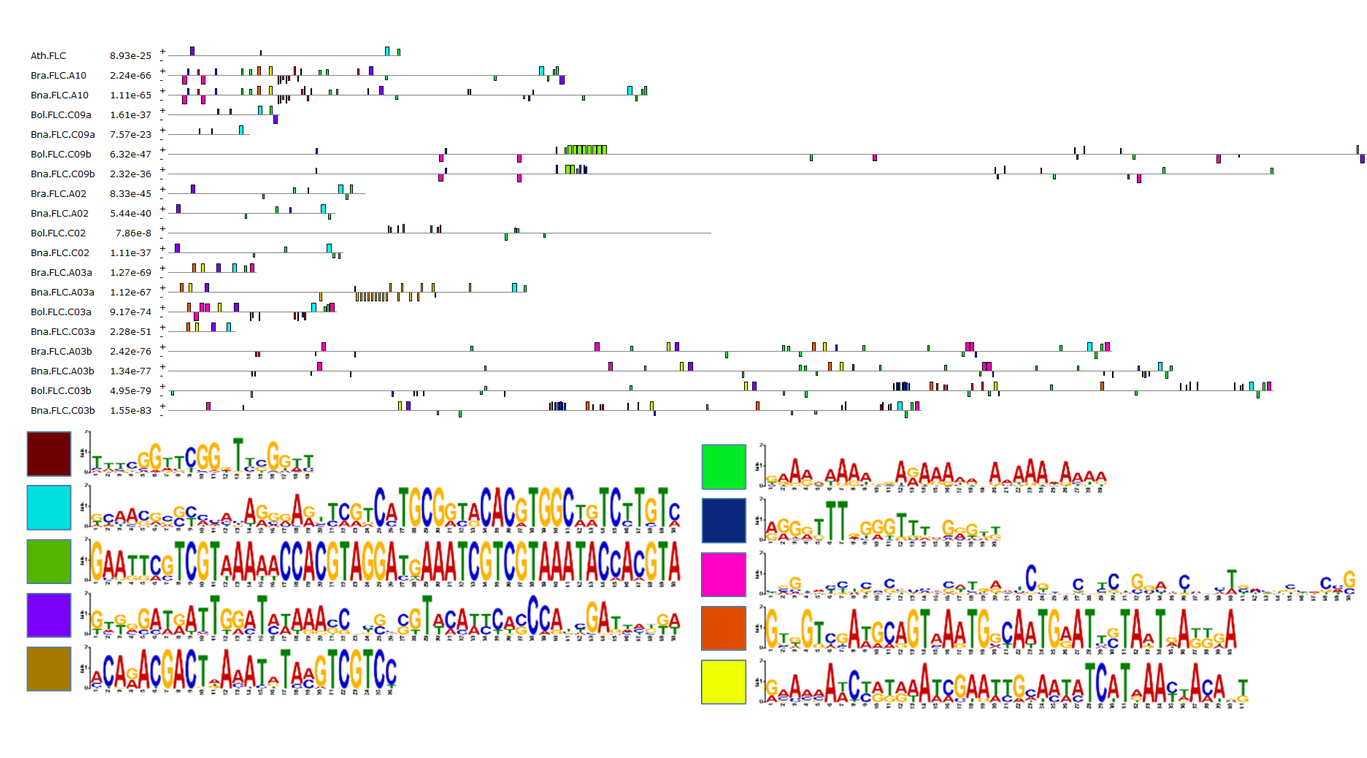


**Figure S5: Motif composition of the upstream (promotor) regions of 19 analysed *FLC* orthologs of *B. rapa, B. oleracea, B. napus* and *A. thaliana* gene copies done by MEME. Every motif is represented by a distinct color (see legend).**


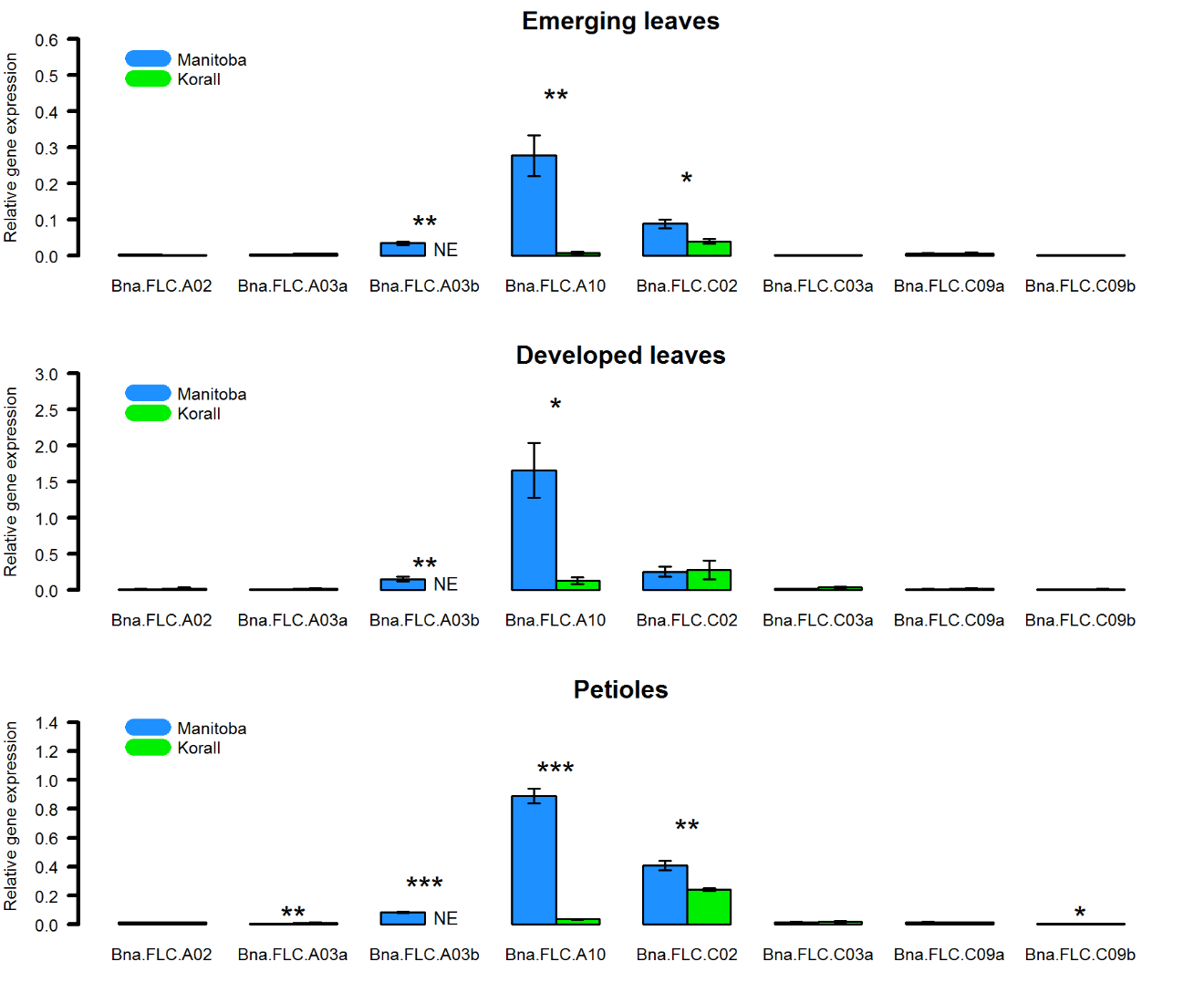


Figure S6: Relative gene expression of all eight expressed *Bna.FLC* copies in emerging and developed leaves and petioles in the accessions Manitoba (winter-type) and Korall (spring-type) without vernalisation at BBCH 15. The values were calculated from RT-qPCR using the ΔCt method and represent 3 biological replicates. Whiskers show SEM. Asterisks show the level of significance based on the Student’s t-test (*p-value<0.05, **p-value<0.01, ***p-value<0.001). “NE” stands for “not expressed”.

**
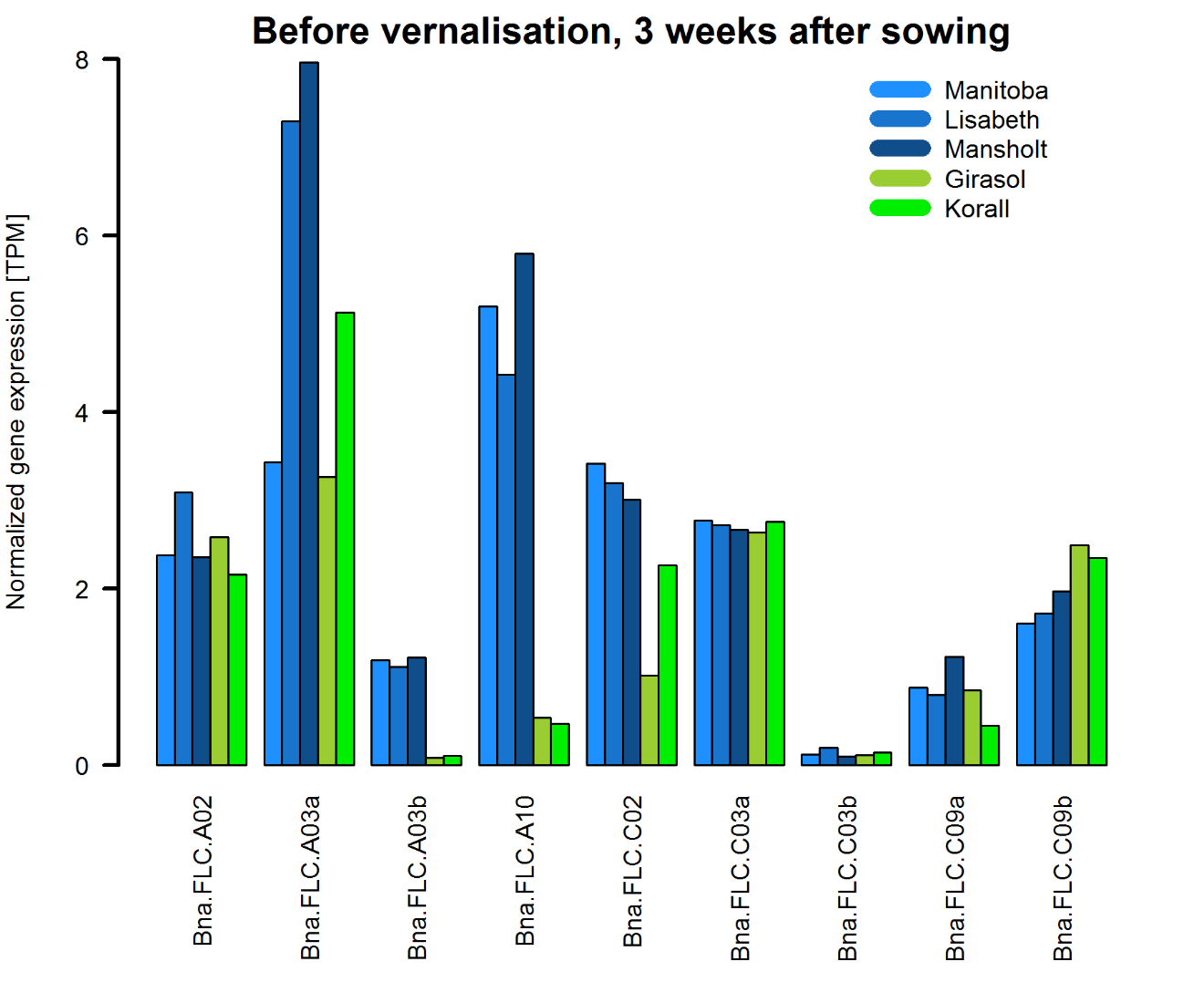
**

**Figure S7: Normalized gene expressed levels from RNAseq data for all 9 annotated *Bna.FLC* copies in the same accessions as used for the RT-qPCR experiment. Blue bars represent winter accessions, green bars represents spring accessions. As no biological replicates are available, no SEM can be computed.**

**
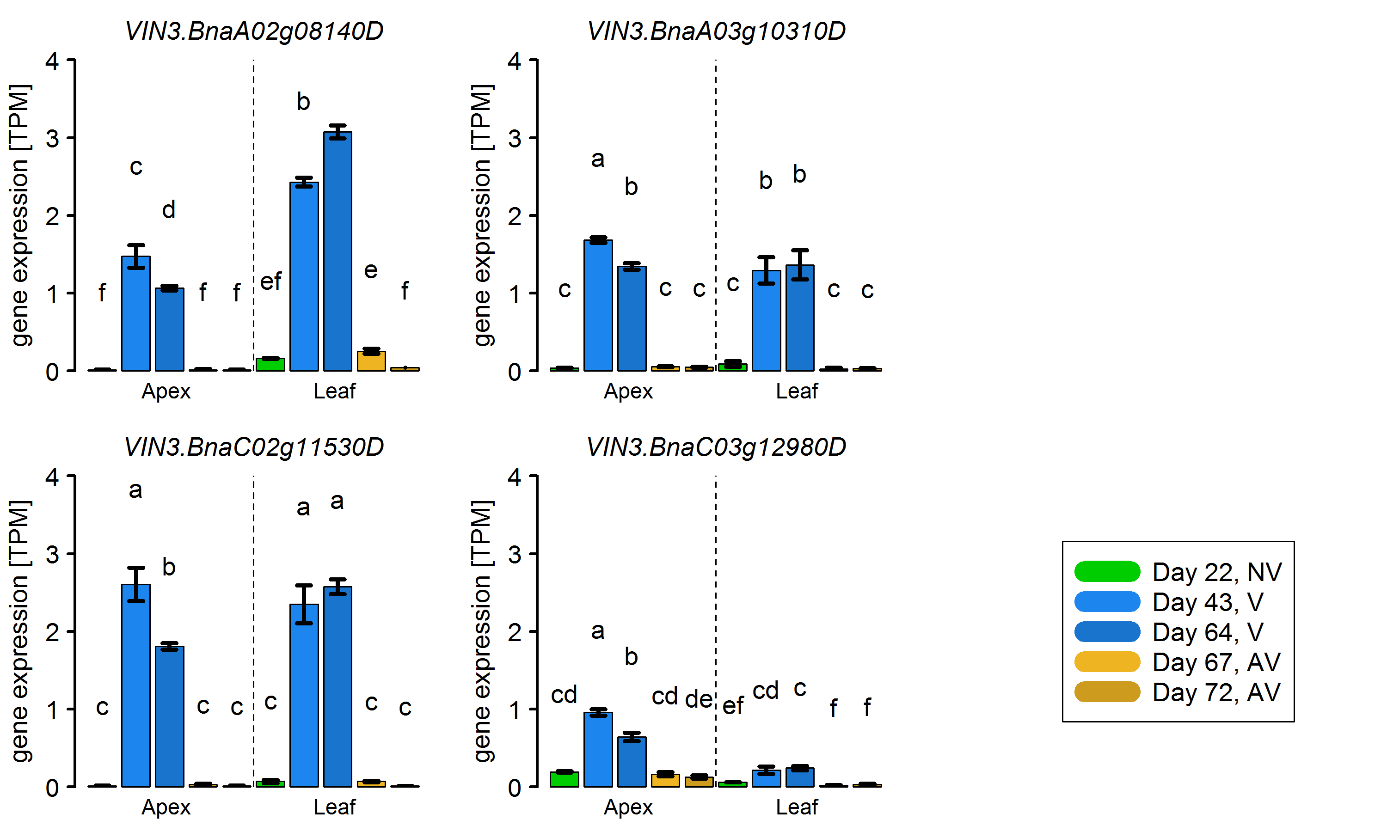
**

**Figure S8: Time series of *Bna.VIN3* expression. Normalized gene expressed levels (TPM) values from RNAseq from a publically available data set for the spring cultivar Westar.**

**
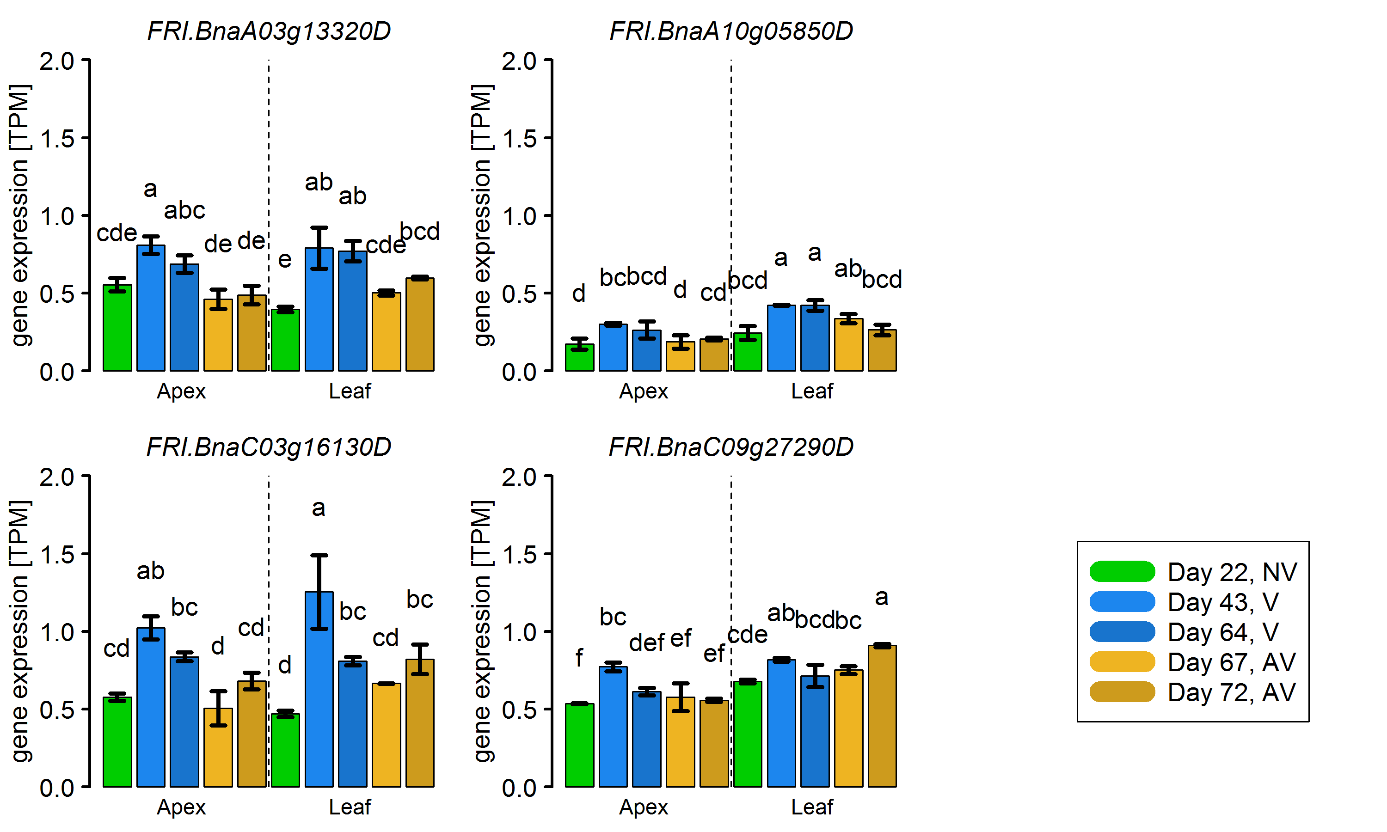
**

**Figure S9: Time series of *Bna.FRI* expression. Normalized gene expressed levels (TPM) values from RNAseq from a publically available data set for the spring cultivar Westar.**

**
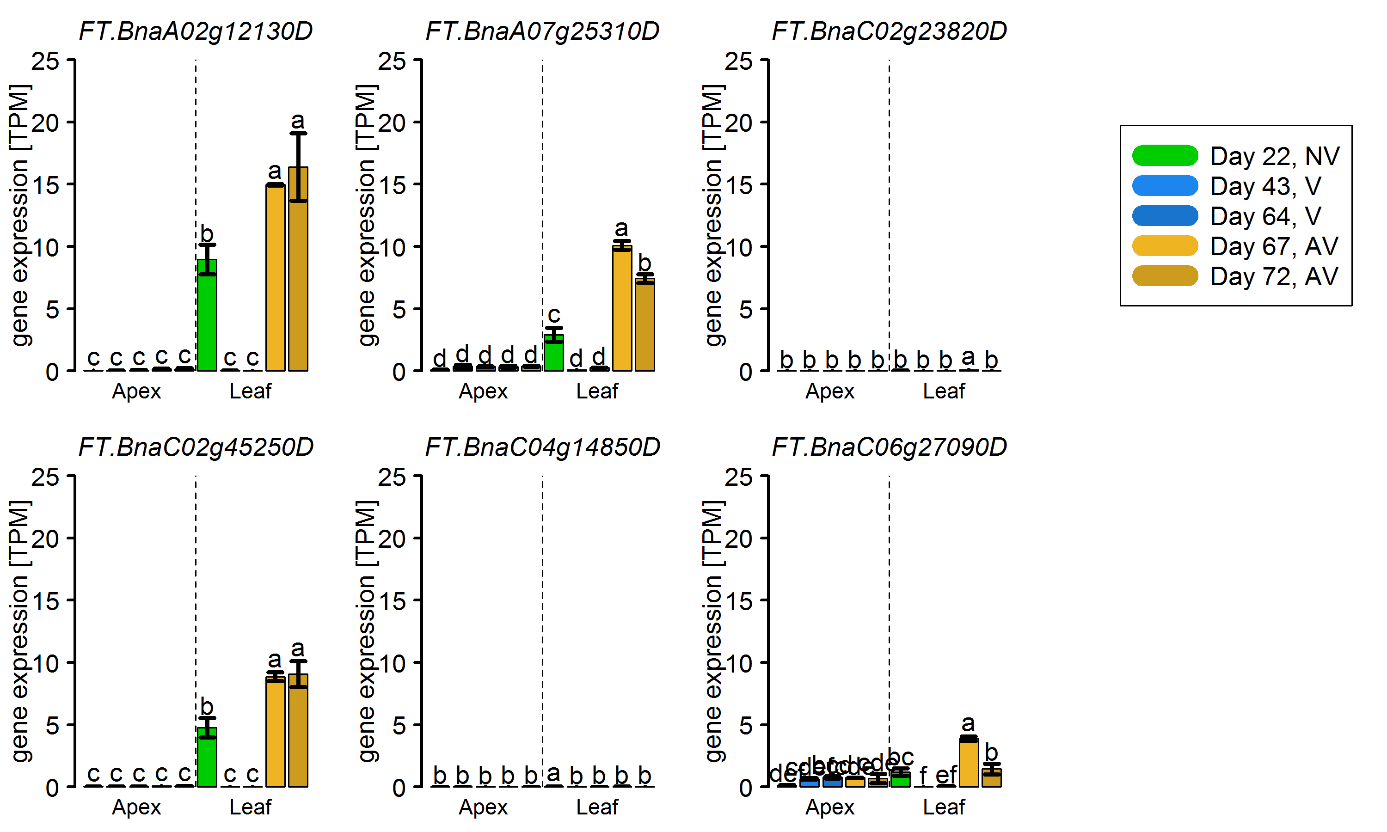
**

**Figure S10: Time series of *Bna.FT* expression. Normalized gene expressed levels (TPM) values from RNAseq from a publically available data set for the spring cultivar Westar.**
